# Effects of Methotrexate Alone or Combined With Arthritis-Related Biotherapies in an *in vitro* Co-culture Model With Immune Cells and Synoviocytes

**DOI:** 10.3389/fimmu.2019.02992

**Published:** 2019-12-20

**Authors:** Mélissa Noack, Pierre Miossec

**Affiliations:** ^1^Immunogenomics and Inflammation Research Unit, Edouard Herriot Hospital, Hospices Civils de Lyon, Lyon, France; ^2^University Claude Bernard Lyon 1, Lyon, France

**Keywords:** methotrexate, biotherapies, rheumatoid arthritis, cell interaction, pro-inflammatory cytokines

## Abstract

**Background:** Methotrexate (MTX) at low dose is a key drug for rheumatoid arthritis (RA). MTX is widely used alone or combined with biologics or steroids. The aim was to study its effects on cytokine production using an *in vitro* model with synoviocytes interacting with peripheral blood mononuclear cells (PBMC) to reproduce the interactions in RA synovium.

**Methods:** Activated-PBMC were co-cultured with RA synoviocytes during 48 h. A dose-response of MTX was tested and different biotherapies (Infliximab, Tocilizumab, Abatacept and Rituximab) were added alone or in combination with MTX. Cytokine production (IL-17, IL-6, IL-1β, IFN-γ, and IL-10) was measured by ELISA. These results were compared with those obtained with steroids.

**Results:** MTX alone had a modest inhibitory effect on cytokine production compared to steroids. The most effective concentration was one of the lowest, 0.01 μg/ml, as for steroids. Infliximab was the most active biotherapy (*p* ≤ 0.05 for all cytokines) followed by Tocilizumab (*p* ≤ 0.05 for all cytokines except IL-6). Abatacept and Rituximab had a more restricted effect on cytokines (*p* ≤ 0.05 for IL-1β and IFN-γ). The combination MTX/biotherapies did not increase significantly the inhibition of cytokine production but some specific inhibitory effects were observed with Infliximab on IL-17 and IL-6, and with Abatacept and Rituximab on IL-1β.

**Conclusion:** Low dose of MTX was at least as effective as high dose. The effects of the combination with biotherapies showed an important level of heterogeneity between the levels of some specific cytokines and the degree of inhibition with drugs.

## Introduction

Methotrexate (MTX) at low dose (1) is a key drug for inflammatory arthritis notably rheumatoid arthritis (RA). MTX is widely used alone or combined with biologics or steroids (2, 3). Developed as a folic acid antagonist, MTX at high dose inhibits dehydrofolate reductase, thus preventing the conversion of dihydrofolate to tetrahydrofolate. This leads to the inhibition of the synthesis of purines and pyrimidines, interfering with DNA synthesis (4, 5). At low dose, part of the anti-inflammatory effect of MTX results from the production of adenosine, with an inhibition of neutrophil function (6). In an *in vivo* model of inflammation, MTX increased adenosine concentration and decreased leukocyte accumulation in inflammatory exudates (7).

In diseases treated with MTX, such as psoriasis or RA, the contribution of local cell-cell interactions is critical in maintaining the chronicity of inflammation. Interactions between infiltrated immune cells and local mesenchymal cells at the inflammatory site lead to high production of pro-inflammatory cytokines, such as IL-6, TNF or IL-17. To reproduce these conditions, we have set up a model of co-culture mimicking the *in vivo* situation (8, 9). In the context of RA, interactions between resting PBMC and synoviocytes were sufficient to induce IL-6 and IL-1β secretion while both PBMC activation and cell interactions were needed to induce a high IL-17 production (8). In the context of psoriasis, similar results were found. Interactions between skin fibroblasts and resting PBMC induced IL-8, IL-6, and IL-1β secretion while a high IL-17 production was found only in co-cultures between skin fibroblasts and activated PBMC (9). Using this model of co-culture between RA synoviocytes and activated PBMC, a previous study has shown the effect of current biotherapies for arthritis combined with corticosteroids (10).

Herein, the aim was to extend this previous study by analyzing with the same model the effect of MTX used alone or in combination with current biotherapies and by comparing the effects of MTX to those of corticosteroids.

## Materials and Methods

### Samples

RA synoviocytes were obtained as previously described (11), from synovial tissue of RA patients undergoing joint surgery and who fulfilled the American College of Rheumatology criteria for RA (12). Synovial tissue was minced into small pieces and then adhered in 6-well plates in Dulbecco's modified Eagle's medium (DMEM; Eurobio, Courtaboeuf, France) supplemented with 10% fetal bovine serum (FBS; Life Technologies, Carlsbad, USA), 2 mM L-glutamine and 100 U/ml penicillin/streptomycin. Cells were maintained at 37°C in a humidified 5% carbon dioxide incubator and used between passages 4 and 9. Synoviocytes used beyond the third passage, were previously characterized by CD45– CD34– CD73– CD90+ CD105+ (13). PBMC from healthy donors were isolated by Ficoll-Hypaque (Eurobio, Courtaboeuf, France) density-gradient centrifugation. Each individual signed an informed consent form. The protocol was approved by a committee for the protection of persons participating in biomedical research (AC-2016-2729).

### Co-culture Assays

Co-culture was initiated by seeding RA synoviocytes overnight in 96-well plates at a density of 2 × 10^4^ cells/well in RPMI 1640 medium (Eurobio, Courtaboeuf, France) supplemented with 10% human AB serum (Blood Bank Center in Lyon, France), 2 mM L-glutamine and 100 U/ml penicillin/streptomycin (complete RPMI), as previously described (11). The next day, PBMC (1 × 10^5^ cells/well) were pre-incubated for 3 h in complete RPMI with or without different treatments and then, without washes, directly seeded at a 5:1 ratio, in the presence of phytohemagglutinin (PHA, 5 μg/ml). After 48 h, supernatants were collected for analysis (13).

### Treatments

A dose-response of Methotrexate (Biodim, Neuraxpharm France, Paris, France) was done with 0, 0.001, 0.01, 0.1, 1, and 10 μg/ml. The concentration of 0.01 μg/ml was used in combination with biotherapies. Based on previous results, the concentration 10 μg/ml of biotherapies was used in co-culture (10). Infliximab (Remicade, anti-TNF, MSD France, Courbevoie, France), Tocilizumab (Roactemra, anti-IL-6 receptor, Roche, Boulogne-Billancourt, France), Abatacept (Orencia, CTLA4 Ig, Bristol-Myers Squibb, Rueil-Malmaison, France), Rituximab (Mabthera, anti-CD20, Roche, Boulogne-Billancourt, France) were tested. A control antibody, IgG1 directed against the BetV1 allergen was used as irrelevant antibody, at the same concentration than biologics (anti-BetV1 Ab, Dendritics, Lyon, France).

### Enzyme-Linked Immunosorbent Assays (ELISAs)

IL-17A, IL-6, IL-1β, IFN-γ, and IL-10 productions were evaluated from culture supernatants with commercially available Duoset ELISA kits, according to the manufacturer's instructions (R&D system, Minneapolis, USA).

### Statistical Analysis

Statistical analyses were performed using paired Wilcoxon test. All analyses were performed with Graph Pad Prism 6 software. *p* ≤ 0.05 were considered as significant.

For correlation analysis, different regressions were tested. The non-linear regression with a second order polynomial was chosen based on the best r square. Then, r was calculated and a none or weak correlation was defined with a r value between −0.5 and 0.0 (negative correlation) and between 0.0 and 0.5 (positive correlation) and a strong correlation was defined with a r value between −1.0 and −0.5 (negative correlation) and between 0.5 and 1 (positive correlation). A linear correlation was done between IL-17 production and IL-6, IL-1β, IFN-γ or IL-10 secretion.

## Results

### Effects of Methotrexate Alone

**Figure 1:**
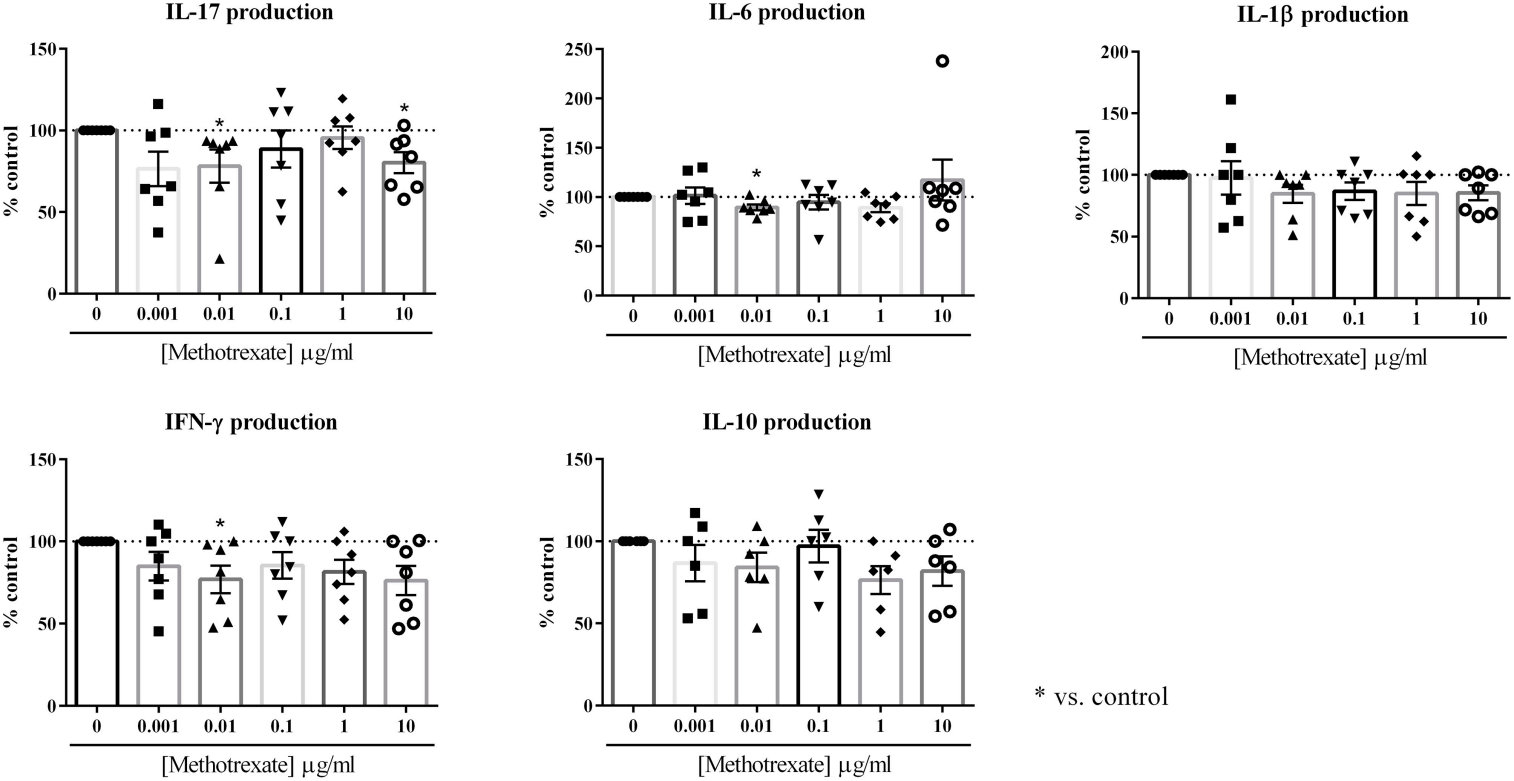
Effect of the dose-response of Methotrexate on cytokine production. Healthy PBMC activated by PHA (5 μg/ml) were pre-incubated or not with different doses of Methotrexate (0, 0.001, 0.01, 0.1, 1, and 10 μg/ml). Then, PBMC were co-cultured with RA synoviocytes at a ratio 5:1 for 48 h. The production of IL-17, IL-6, IL-1β, IFN-γ, and IL-10 in cell supernatants was measured by ELISA. *p ≤ 0.05. Results are represented as mean ± SEM, *n* = 7 experiments from five RA patients. Synoviocytes or PBMC alone produced very low or undetectable levels of cytokines in control condition (IL-17, IFN-γ, and IL-10: undetectable for both cell types; IL-1β: undetectable for synoviocytes; 7.2 ± 16.1 pg/ml for PBMC; IL-6: 6.1 ± 2.3 ng/ml for synoviocytes; 1.4 ± 3.4 ng/ml for PBMC).

The aim was to evaluate its effect in an *in vitro* co-culture model, between RA synoviocytes and PBMC. As previously described (10), this model aims to mimic the cell interactions which occur at the inflammatory site between mesenchymal and immune cells, leading to pro-inflammatory cytokines. Indeed, cell interactions between activated-PBMC and RA synoviocytes lead to a major increase in cytokine production, notably for IL-17. This effect is mainly due to cell-cell interactions and not to soluble factors, as demonstrated by using a transwell system (8). Its relevance was further validated by using an autologous system, with PBMC and synoviocytes from the same RA patient (8). A dose-response curve was performed, with different concentrations, 0, 0.001, 0.01, 0.1, 1, and 10 μg/ml. The effect of MTX was evaluated on the production of several pro- and anti-inflammatory cytokines, IL-17, IL-6, IL-1β, IFN-γ, and IL-10, after 48 h of culture. The concentration of these cytokines was measured by ELISA in the supernatants. The results in Figure 1 were expressed as the percentage of each cytokine production with drugs compared to the control condition (100 %).

For IL-17, a significant decrease was observed only at the concentrations of 0.01 μg/ml and 10 μg/ml (78.1 and 80.3%, respectively, *p* ≤ 0.05, Figure 1). The intermediate concentrations had no significant effect (0.1 μg/ml: 88.6% and 1 μg/ml: 95.4%, Figure 1).

For IFN-γ, as for the other cytokines, no dose-response was observed. The most effective concentration was 0.01 μg/ml that decreased significantly IFN-γ production (76.9%, *p* ≤ 0.05, Figure 1). The other concentrations decreased the IFN-γ secretion between 15 and 25% but it was not significant (0.001 μg/ml: 85.0%; 0.1 μg/ml: 85.5%; 1 μg/ml: 81.5% and 10 μg/ml: 76.2%, *p* > 0.05, Figure 1).

For IL-6, only the concentration of 0.01 μg/ml induced a significant decrease of secretion (89.5%, *p* ≤ 0.05, Figure 1). For IL-1β, the lower concentration of 0.001 μg/ml had no effect (97.5%, Figure 1), while with all the others, a 15% decrease was observed from 0.01 to 10 μg/ml (0.01 μg/ml: 84.7%, 0.1 μg/ml: 86.8%, 1 μg/ml: 84.9% and 10 μg/ml: 85.4%, Figure 1), but this decrease was not significant.

For IL-10 secretion, no significant decrease was induced by MTX, even if the production was reduced to 25% (0.001 μg/ml: 86.7%; 0.01 μg/ml: 84.1%; 0.1 μg/ml: 97.1%; 1 μg/ml: 76.4% and 10 μg/ml: 81.7%, *p* > 0.05, Figure 1).

In conclusion, MTX alone had a rather modest effect on cytokine production during cell interactions. No clear dose-response was observed and the most effective concentration was 0.01 μg/ml. This low concentration is in line with the most effective concentration of methylprednisolone determined in our previous experiments (10). Thus, this concentration of 0.01 μg/ml was kept for the experiments of MTX combined with biotherapies.

### Comparison Between Different Biotherapies

The most common biotherapies used in RA included biotherapies targeting cytokines, such as Infliximab, a monoclonal antibody which blocks TNF or Tocilizumab, a monoclonal antibody which blocks the IL-6 receptor, and biotherapies targeting cells, such as Abatacept, a fusion protein (CTLA-4-Ig) which interacts with B7 (ligand of CD28) or Rituximab, a monoclonal antibody against CD20. Here, these four different biotherapies were used in the *in vitro* co-culture model to test and compare their effects on cytokine production. The lowest effective concentration of 10 μg/ml, defined in our previous work (10), was used.

**Figure 2:**
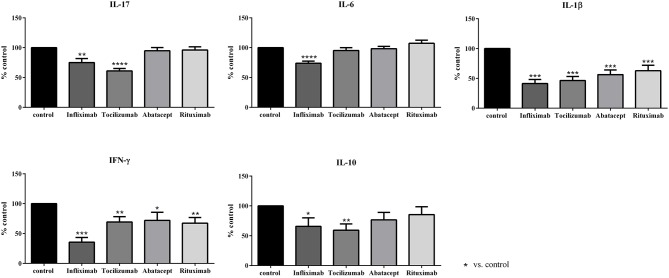
Effect of biotherapies on cytokine production. Healthy activated-PBMC were pre-incubated or not with biotherapies at 10 μg/ml and co-cultured with RA synoviocytes at a ratio 5:1 for 48 h. Infliximab, Tocilizumab, Abatacept and Rituximab were tested. After 48 h, the production of IL-17, IL-6, IL-1β, IFN-γ, and IL-10 in cell supernatants was measured by ELISA. *p ≤ 0.05. Results are represented as mean ± SEM, *n* = 15 experiments from six RA patients. The use of a control antibody at the same concentration as biologics had no effect in the assays and gave similar results as control (data not shown).

The results were presented in Figure 2. The cytokine production in control condition was established at 100% and the results were expressed as percentage of the control production.

For IL-17, only Infliximab and Tocilizumab induced a significant decrease of IL-17 secretion (75.0 and 61.1%, respectively, *p* ≤ 0.05), with a higher effect for Tocilizumab. Abatacept and Rituximab did not have an effect on IL-17 production (94.8 and 96.2%, respectively, Figure 2).

IL-6 secretion was reduced only by Infliximab that decreased significantly IL-6 production (74.0%, *p* ≤ 0.05) while the three others had no effect (95.4% for Tocilizumab; 98.3% for Abatacept; 107.3% for Rituximab, Figure 2).

IL-1β was the cytokine the most affected by all biotherapies. Its secretion was decreased by about 50 % (Infliximab, 38.9%; Tocilizumab, 43.5%; Abatacept, 52.8%; Rituximab, 58.9%, *p* ≤ 0.05, Figure 2).

The secretion of IFN-γ was mostly inhibited by Infliximab (33.1%, *p* ≤ 0.05). Tocilizumab, Abatacept and Rituximab had a similar inhibitory effect on IFN-γ production (72.5, 67.1, and 62.8%, respectively, *p* ≤ 0.05, Figure 2).

The anti-inflammatory cytokine IL-10 was also affected by biotherapies. Infliximab and Tocilizumab inhibited significantly its secretion (65.7 and 59.3%, respectively, *p* ≤ 0.05, Figure 2) while the decrease induced by Abatacept and Rituximab was not significant (76.7 and 85.5%, respectively, Figure 2).

As a summary, Infliximab was the most active biotherapy, inhibiting a large panel of cytokines (IL-17, IL-6, IL-1β, IFN-γ, and IL-10), followed by Tocilizumab. These two biotherapies target directly cytokines and their receptors possibly explaining their broad effect on cytokines. Conversely, Abatacept and Rituximab that target specific cells had a more restricted effect on cytokines compared to Infliximab and Tocilizumab.

### Combination of MTX and Biotherapies

MTX is frequently more active in combination with biologics. To better understand the impact of these combinations, the effect of MTX combined to biotherapies was tested in our *in vitro* model of cell interactions. Four current biotherapies used in RA treatment have been chosen for the combination study: two biotherapies targeting directly cytokine or receptor, Infliximab and Tocilizumab, and two biotherapies targeting cells, Abatacept and Rituximab. The different mechanisms of action of biologics could allow a better understanding of the different responses. Then, the results were compared to the effects of steroids in combination or not with those biotherapies as in a previous study (10). This study showed that high doses of steroids were not more active than low doses. The lowest effective concentration of methylprednisolone (MP) was also 0.01 μg/ml, as for MTX alone.

**Figure 3:**
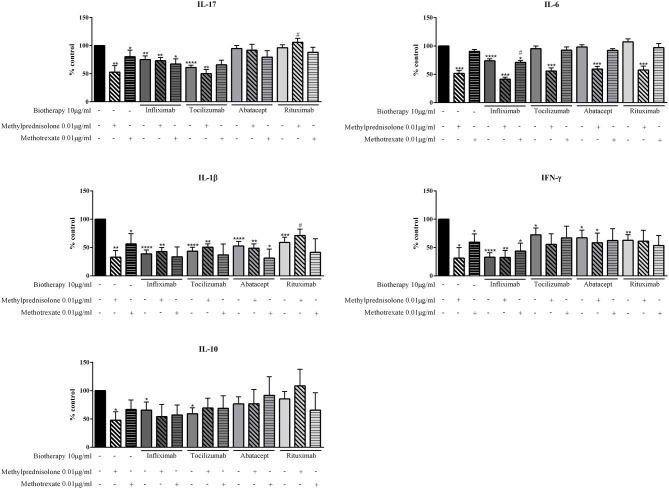
Effect of the combination of Methotrexate or Methylprednisolone and biotherapy on pro-inflammatory cytokine production. Healthy activated-PBMC were pre-incubated or not with Methotrexate alone (0.01 μg/ml), biotherapy alone (10 μg/ml), or with the combination of both treatments. Then, PBMC were co-cultured with RA synoviocytes at a ratio 5:1 for 48 h. Infliximab, Tocilizumab, Abatacept and Rituximab were tested. After 48 h, the production of IL-17, IL-6, IL-1β, and IFN-γ in cell supernatants was measured by ELISA. Data from our previous study were used for methylprednisolone (MP) results (10). *, #*p* ≤ 0.05. *compares with control and # compares combination MTX/biotherapy or MP/biotherapy vs. MTX alone or MP alone, respectively. Results are represented as mean ± SEM, *n* = 6 experiments from five RA patients for results with MTX and *n* = 11 experiments from six RA patients for results with MP.

The comparison between MTX and MP alone indicated that MP was more efficient to reduce cytokine production. MP decreased at least by half cytokine production (IL-17: 52.9%; IL-6: 51.6%; IL-1β: 32.9%; IFN-γ: 31.4%; IL-10: 47.8%, *p* ≤ 0.05, Figure 3) while MTX had a significant but lower effect on IL-17, IL-1β, and IFN-γ (IL-17: 80.1%; IL-1β: 56.3%; IFN-γ: 59.4%, *p* ≤ 0.05, Figure 3) and slightly decreased IL-6 secretion (90.5%, *p* = 0.06, Figure 3). For IL-10, MTX decreased its secretion but without reaching a significant effect (66.8%, *p* = 0.06, Figure 3).

The effect of the combination of MTX or MP and biotherapies was complex to analyze with an effect depending on each cytokine. Regarding first the combination of MP and biotherapies, for IL-17, the combination of MP with Infliximab, Abatacept or Rituximab did not have an additional effect of inhibition, even more the inhibitory effect of MP was rather reduced with the biotherapies (MP: 52.9%; Infliximab: 75.0%; Infliximab+MP: 73.1%; Abatacept: 94.8%; Abatacept+MP: 91.8%; Rituximab: 96.2%; Rituximab+MP: 105.9%, Figure 3). With Tocilizumab, the inhibition of IL-17 secretion was similar to MP alone (MP: 52.9%; Tocilizumab: 61.1%; Tocilizumab+MP: 50.2%, Figure 3).

The combinations with MTX induced different effects than those with MP on IL-17 secretion. With Tocilizumab and Rituximab, the inhibition was similar to the inhibition by each biotherapy alone (MTX: 80.1%; Tocilizumab: 61.1%; Tocilizumab+MTX: 65.8%; Rituximab: 96.2%; Rituximab+MTX: 88.1%, Figure 3). With Abatacept, the inhibition was similar to MTX alone (MTX: 80.1%; Abatacept: 94.8%; Abatacept+MTX: 79.5%). With Infliximab, a modest additional inhibitory effect was observed (MTX: 80.1%; Infliximab: 75.0%; Infliximab+MTX: 67.3%, Figure 3).

For IL-6, the combination of MP with Tocilizumab, Abatacept or Rituximab had no additional inhibitory effect than MP alone (MP: 51.6%; Tocilizumab: 95.6%; MP+Tocilizumab: 55.8%; Abatacept: 98.3%; MP+Abatacept: 59.3%; Rituximab: 107.3%; MP+Rituximab: 57.7%, Figure 3). A modest additional inhibition was observed with Infliximab (MP: 51.6%; Infliximab: 74.0%; MP+Infliximab: 41.5%, Figure 3). With MTX, the combination with Tocilizumab, Abatacept or Rituximab, as for MP, induced the same inhibition as MTX alone (MTX: 90.5%; Tocilizumab: 95.6%; MP+Tocilizumab: 55.8%; Abatacept: 98.3%; MP+Abatacept: 59.3%; Rituximab: 107.3%; MP+Rituximab: 97.1%, Figure 3). For the combination MTX/Infliximab, the inhibition was similar to Infliximab alone (MTX: 90.5%; Infliximab: 74.0%; MTX+Infliximab: 71.0%, Figure 3).

For IL-1β, the combination of Infliximab with MP or MTX and Tocilizumab with MP or MTX induced a production similar to the level with biotherapy alone (MP: 32.9%; MTX: 56.3%; Infliximab: 38.9%; Infliximab+MP: 43.1%; Infliximab+MTX: 33.5%; Tocilizumab: 43.5%; Tocilizumab+MP: 40.5%; Tocilizumab+MTX: 37.0%, Figure 3). Nevertheless, the level of IL-β with biotherapy was higher than MP alone while it was lower than MTX alone. Abatacept in combination with MP induced a similar IL-1β inhibition than Abatacept alone, which was weaker than MP alone (MP: 32.9%; Abatacept: 52.8%; MP+Abatacept: 48.8%, Figure 3). The combination Abatacept/MTX had an additional inhibitory effect on IL-1β production (MTX: 56.3%; Abatacept: 52.8%; MTX+Abatacept: 31.3%, Figure 3). For Rituximab, the combination with MP or MTX had an opposite effect. With Rituximab/MP, the IL-1β inhibition was weaker than with MP or Rituximab alone (MP: 32.9%; Rituximab: 58.9%; MP+Rituximab: 71.3%, Figure 3) while for Rituximab/MTX, the secretion of IL-1β was inhibited stronger than with MTX or Rituximab alone (MTX: 56.3%; Rituximab: 58.9%; MTX+Rituximab: 41.6%, Figure 3).

For IFN-γ, the combination Infliximab/MP had no additional inhibitory effect compared to Infliximab or MP alone, inducing a similar production of IFN-γ (MP: 31.4%; Infliximab: 33.1%; MP+Infliximab: 32.6%, Figure 3). With Infliximab/MTX, the inhibition of IFN-γ production was lower than Infliximab alone but higher than MTX alone (MTX: 59.4%; Infliximab: 33.1%; MTX+Infliximab: 43.8%). Tocilizumab and Abatacept decreased the inhibitory effect of MP alone but the combination still induced a higher inhibition than Tocilizumab or Abatacept alone (MP: 31.4%; Tocilizumab: 72.5%; Tocilizumab+MP: 55.7%; Abatacept: 67.1%; Abatacept+MP: 58.4%, Figure 3). With MTX, the combination with Tocilizumab or Abatacept induced similar effect than with MP, but to a lower extent (MTX: 59.4%; Tocilizumab: 72.5%; Tocilizumab+MTX: 67.0%; Abatacept: 67.1%; Abatacept+MTX: 62.4%, Figure 3). With Rituximab, the inhibitory effect of MP was fully blocked as the IFN-γ level was similar to Rituximab alone (MP: 31.4%; Rituximab: 62.8%; MP+Rituximab: 61.2%) while a modest additional inhibitory effect was observed with MTX (MTX: 59.4%; Rituximab: 62.8%; MTX+Rituximab: 53.5%, Figure 3).

For IL-10, the inhibitory effect of MP was reversed by all biotherapies as the level of IL-10 was lower than control but higher than MP alone. With Infliximab/MP, the inhibitory effect was still higher than Infliximab alone (MP: 47.8%; Infliximab: 65.7%; Infliximab+MP: 54.4%, Figure 3) while with Abatacept/MP, IL-10 inhibition was similar as Abatacept alone (MP: 47.8%; Abatacept: 76.7%; Abatacept+MP: 76.8%, Figure 3). The combinations Tocilizumab/MP and Rituximab/MP induced an inhibitory effect on IL-10 lower than biotherapies alone (MP: 47.8%; Tocilizumab: 59.3%; Tocilizumab+MP: 69.6%; Rituximab: 85.5%; Rituximab+MP: 108.4% Figure 3). For MTX, this weaker inhibition of IL-10 level was observed only with the combination Abatacept/MTX (MTX: 66.8%; Abatacept: 76.7%; MTX+Abatacept: 91.7%, Figure 3). For Tocilizumab and Rituximab, the combination with MTX induced an inhibitory effect similar to MTX alone while a slight additional inhibitory effect was observed with Infliximab/MTX (MTX: 66.8%; Tocilizumab: 59.3%; MTX+Tocilizumab: 68.8%; Rituximab: 85.5%; MTX+Rituximab: 65.7%; Infliximab: 65.7%; MTX+Infliximab: 57.1%, Figure 3).

In summary for this part, the combination MP/biotherapies did not induce a higher effect than MP alone. For IL-17, IL-1β, IFN-γ, and IL-10, the combination induced a rather similar effect than biotherapies alone while for IL-6 the inhibitory effect of MP alone was kept. The inhibitory effect of MTX was lower than that of MP. Despite some additional specific inhibitory effects observed mainly with Infliximab on IL-17 and IL-6, and with Abatacept and Rituximab on IL-1β, the combination MTX/biotherapies did not increase significantly the inhibition of cytokine production.

### Correlation Between Cytokine Productions

**Figure 4:**
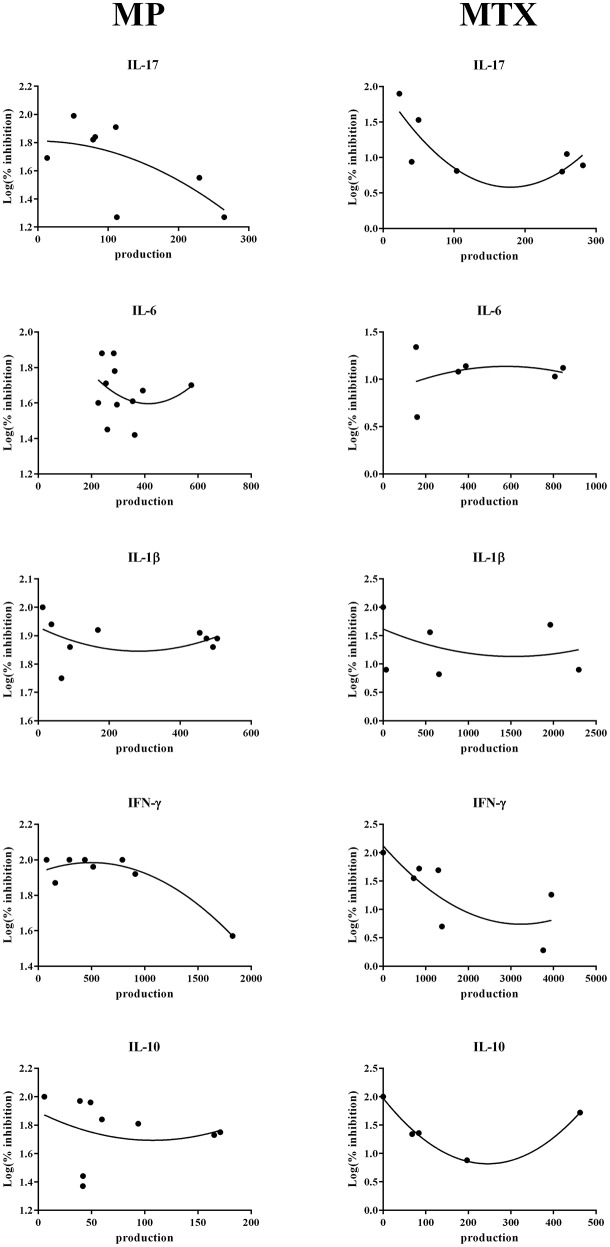
Correlation between cytokine production and inhibition by Methotrexate or Methylprednisolone. Using the levels measured in these experiments, correlations were calculated between the level of cytokine production and the logarithm of the percentage of inhibition with Methotrexate or Methylprednisolone. Results were represented by a second order polynomial non-linear regression.

The strong heterogeneity observed in the treatment response in patients could be a consequence of individual variations in cytokine production. Using the levels measured in these experiments, correlations were calculated between the level of cytokine production and the logarithm of the percentage of inhibition with MTX or MP. After different attempts, the best mode of calculation was a second order polynomial non-linear regression (Figure 4). No correlation was found for IL-6 and IL-1β for MTX and for MP (IL-6: *r* = 0.24 for MTX and *r* = −0.29 for MP; IL-1β: *r* = −0.36 for MTX and *r* = −0.27 for MP). For IL-10, the level of production could be related only with the effect of MTX but not with that of MP (*r* = −0.99 for MTX and *r* = −0.2 for MP). For IFN-γ and IL-17, a negative correlation was observed for both MTX and MP (IFN-γ: *r* = −0.76 for MTX and *r* = −0.95 for MP; IL-17: *r* = −0.79 for MTX and *r* = −0.65 for MP). Thus, a high production of IL-17 and IFN-γ induced a lower percentage of inhibition for MTX and for MP.

**Figure 5:**
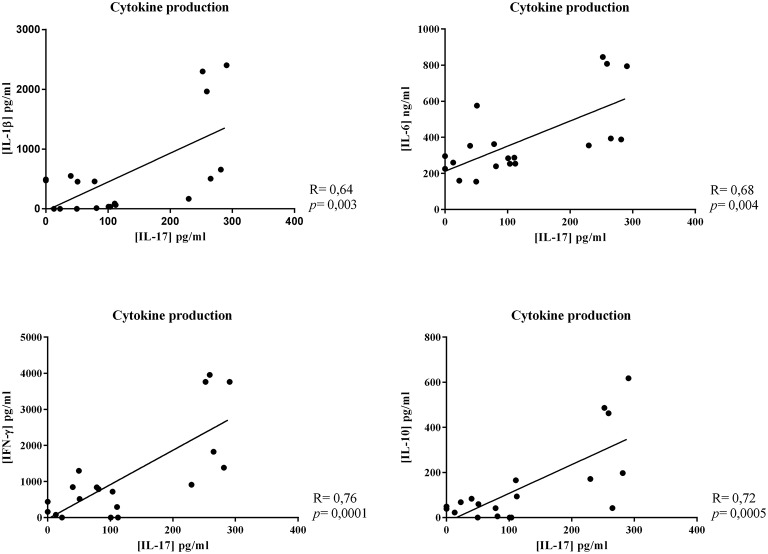
Correlation between cytokine productions. Linear correlations between IL-17 production and the other cytokine production were calculated. Results represented this linear correlation between IL-17 production and IL-1β, IL-6, IFN-γ, or IL-10 production.

Focusing on IL-17, linear correlations between IL-17 production and the other cytokine production were calculated. A strong positive correlation was observed between IL-17 production and that of all the other cytokines (IL-17/IL-1β, *r* = 0.64; IL-17/IL-6, *r* = 0.68; IL-17/IFN-γ, *r* = 0.76; IL-17/IL-10, *r* = 0.72, Figure 5).

Finally, a high production of IL-17 was also correlated with a lower percentage of inhibition, mainly by MTX.

## Discussion

The aim of this work was to study the effects of MTX vs. those of steroids on cytokine production using an *in vitro* cell interaction model (14). MTX was used alone or in combination with current RA biotherapies.

The *in vitro* model is based on interactions between synoviocytes and activated immune cells to mimic the local *in vivo* inflammatory state. The effects of MTX were studied first with a dose-response. For each tested cytokine (IL-17, IL-6, IL-1β, IFN-γ, and IL-10), no clear dose-response was observed and MTX alone had a modest inhibitory effect on such cytokine secretion. For steroids, a large anti-inflammatory effect was shown and a dose-effect was observed on the inhibition of IL-17, IL-6, and IL-1β while for IFN-γ and IL-10, the maximum effect was reached with the lowest concentration of 0.001 μg/ml (10). Furthermore, the most effective concentration of MTX was not the highest of 10 μg/ml but rather one of the lowest, 0.01 μg/ml. This result was in line with our previous study using steroids in which the lowest effective concentration was also 0.01 μg/ml (10).

The different results obtained between MTX and steroids in these experiments could come from the different modes of action of the two drugs. One key mechanism of action of MTX highlighted for RA is the induction of adenosine signaling. In an *in vivo* model of inflammation, MTX increased adenosine concentration and decreased leukocyte accumulation (7). Furthermore, purinergic G-protein-coupled receptors are overexpressed in RA, allowing a paracrine signal of adenosine. Adenosine can downregulate TNF and NF-κB signaling but it is also involved in the downregulation of the activation and proliferation of T cells (15). Steroids have a completely different mode of action. They diffuse through the cell membrane and exert their functions by binding a glucocorticoid receptor (GR) (16). Steroid effects were mainly genomic effects, with three main mechanisms: the first and the best-described is transactivation; the second is the binding of GR with another transcription factor to a composite element; and the third is the tethering to other transcription factors (14, 16). The large anti-inflammatory effect of steroids compared to MTX was in line with their mechanisms of action that affect several pathways.

Furthermore, in our co-culture model, we have demonstrated that 60% of the high IL-17 secretion resulting from cell interactions is mediated by podoplanin, a type I transmembrane protein (8). In this study, it could be interesting to check the effect of MTX and steroids on podoplanin expression.

In RA patients, a large heterogeneity is present in cytokine production and in response to MTX or steroids. Herein, a correlation was established between the IL-17 and IFN-γ secretion and the efficiency of both MTX and steroids. This information could be used to predict the treatment response of the patients, notably to MTX. A recent study has shown that patients with a higher baseline disease activity and positive for rheumatoid factors had an increased risk to fail MTX treatment (17). This could be explained by our results showing a correlation between a high IL-17 and IFN-γ production and a weak percentage of inhibition of inflammation by MTX. In addition, a recent study showed that specific gene expression profiles could be associated with a higher chance of achieving sustained drug-free remission (18). Furthermore, inflammation induces post translational modifications of proteins, notably citrullination and carbamylation, as in ACPA (anti-citrullinated protein antibodies) and AMPA (anti-modified protein antibodies), with changes in antigenicity (19). All these parameters could explain the strong heterogeneity in treatment response.

It is clear that steroids inhibit pro-inflammatory cytokine production (20), as also shown in our previous work (10), but the effect of MTX on cytokine secretion is more obscure. In an *in vitro* culture of activated immune cells, MTX had a large inhibitory effect on cytokines produced by monocytes (21). In an *in vivo* model of RA, collagen-induced arthritis (CIA), MTX modulated TNF secretion and prevented experimental murine CIA but without effect on IL-6, IL-1β or IFN-γ production (22). In contrast, a recent study has shown that MTX could even induce IL-6 and IL-1β production by a monocytic cell line (23). The fact that MTX could induce pro-inflammatory cytokine may be a reason why combining MTX and biologics has been reported to increase efficacy than a single agent (24). Several clinical trials have shown that the combination of MTX and biologics such as anti-TNF, anti-IL-6 receptor, anti-CTLA-4 or anti-CD20, is more efficacious than MTX alone (3, 25–28).

In this work, our *in vitro* model of co-culture was used to study the combination effect of MTX and biotherapies on cytokine production. Four biotherapies, Infliximab, a monoclonal antibody which blocks TNF, Tocilizumab, a monoclonal antibody which blocks the IL-6 receptor, Abatacept, a fusion protein (CTLA-4-Ig) which interacts with B7 (ligand of CD28) and Rituximab, a monoclonal antibody against CD20, were used. Furthermore, the use of previous data on combination of steroids (use of methylprednisolone, MP) and biotherapies (10) allowed us to compare MTX and MP combination effects. First, the inhibitory effect of MTX was lower than that of MP alone. Then, as previously described, the combination MP/biotherapies did not induce a higher effect than MP alone. Addition of a biotherapy seemed to reduce the effect of MP alone. For MTX, despite some specific inhibitory effects observed mainly with Infliximab on IL-17 and IL-6, and with Abatacept and Rituximab on IL-1β, the combination MTX/biotherapies did not increase significantly the inhibition of cytokine production.

In conclusion, in part through their different mechanisms of action, the effects of MTX and steroids on cytokine production during cell interactions are highly heterogeneous. In both cases, a low dose is at least as effective as a high dose. The effects of the combination with biotherapies are more obvious with those inhibiting cytokines. An important level of heterogeneity is found between the levels of certain specific cytokines and the degree of inhibition with drugs. This may contribute to the level of heterogeneity of response to any drug in the clinic.

## Data Availability Statement

The datasets generated for this study are available on request to the corresponding author.

## Ethics Statement

The studies involving human participants were reviewed and approved by the committee for the protection of persons participating in biomedical research (AC-2016-2729). The patients/participants provided their written informed consent to participate in this study.

## Author Contributions

MN carried out the experiments and drafted the manuscript. PM conceived the study, and reviewed the manuscript. All authors read and approved the final manuscript.

### Conflict of Interest

The authors declare that the research was conducted in the absence of any commercial or financial relationships that could be construed as a potential conflict of interest.
